# Increased staining for phospho-Akt, p65/RELA and cIAP-2 in pre-neoplastic human bronchial biopsies

**DOI:** 10.1186/1471-2407-5-155

**Published:** 2005-12-06

**Authors:** Jay W Tichelaar, Yu Zhang, Jean C leRiche, Paul W Biddinger, Stephen Lam, Marshall W Anderson

**Affiliations:** 1 Department of Environmental Health, University of Cincinnati College of Medicine, Cincinnati, OH, 45267 USA; 2 Department of Genome Science, University of Cincinnati College of Medicine, Cincinnati, OH, 45237 USA; 3 Cancer Imaging Department, British Columbia Cancer Agency, Vancouver, BC, Canada V5Z4E6. USA; 4 Department of Pathology and Laboratory Medicine, University of Cincinnati College of Medicine, Cincinnati, OH, 45267. USA

## Abstract

**Background:**

The development of non-small cell lung carcinoma proceeds through a series of well-defined pathological steps before the appearance of invasive lung carcinoma. The molecular changes that correspond with pathology changes are not well defined and identification of the molecular events may provide clues on the progression of intraepithelial neoplasia in the lung, as well as suggest potential targets for chemoprevention. The acquisition of anti-apoptotic signals is critical for the survival of cancer cells but the pathways involved are incompletely characterized in developing intra-epithelial neoplasia (IEN).

**Methods:**

We used immunohistochemistry to determine the presence, relative levels, and localization of proteins that mediate anti-apoptotic pathways in developing human bronchial neoplasia.

**Results:**

Bronchial epithelial protein levels of the phosphorylated (active) form of AKT kinase and the caspase inhibitor cIAP-2 were increased in more advanced grades of bronchial IEN lesions than in normal bronchial epithelium. Additionally, the percentage of biopsies with nuclear localization of p65/RELA in epithelial cells increased with advancing pathology grade, suggesting that NF-κB transcriptional activity was induced more frequently in advanced IEN lesions.

**Conclusion:**

Our results indicate that anti-apoptotic pathways are elevated in bronchial IEN lesions prior to the onset of invasive carcinoma and that targeting these pathways therapeutically may offer promise in prevention of non-small cell lung carcinoma.

## Background

Lung cancer is the leading cause of cancer mortality in both men and women in the United States [1]. Non-small cell lung carcinomas arise from the respiratory epithelium and progress through well-defined pathological stages prior to becoming invasive and metastatic tumors. While many studies have identified lung tumor markers of clinical or prognostic significance, survival rates for this deadly disease have remained essentially unchanged for the past 30 years. The slow advance in treating lung cancer is due in part to continued gaps in our understanding of the molecular mechanisms of lung tumorigenesis. Thus, studies that aid in our understanding of molecular mechanisms of lung tumorigenesis are important steps towards developing better detection, prevention and treatment of this disease.

Evasion of apoptosis by tumor cells is a critical step during tumorigenesis. The serine/threonine kinase Akt is a critical mediator of anti-apoptotic signaling in eukaryotic cells and is activated in a signaling cascade downstream of Ras activation and phosphoinositide-3-kinase (PI3K) [2]. Amplification of PI3K is common in many tumor types, including lung cancer [3-5] and in lung cancer is correlated with increased phosphorylation of Akt [4]. Activation of Akt, as measured by phosphorylation of the protein, is also increased in multiple tumor types including lung cancer [6-10]. Increased phosphorylation of Akt kinase has also been reported in developing bronchial hyperplasias and dysplasias [7,11,12] and pre-neoplastic atypical alveolar hyperplasia [13], indicating that activation of this pro-survival pathway may be a relatively early event in lung tumorigenesis.

The NF-κB transcription factor family can stimulate both pro- and anti-apoptotic signals. Many studies have described a critical role for NF-κB activity in promoting cell survival. Increased staining for NF-κB subunits has been detected in breast [14] and cervical carcinoma [15]. Inhibition of NF-κB activity either pharmacologically or genetically can sensitize tumor cells to pro-apoptotic agents [16-19] or to tumor necrosis factor-α (TNF-α) induced apoptosis [20]. Proteasome inhibition, which blocks the degradation of inhibitor of κB (IκB) protein, thus blocking NF-κB nuclear translocation and activation, also sensitized NSCLC cells to apoptosis [21,22]. Similarly, expression of a super-repressor form of IκBα sensitized lung cancer cell lines to apoptosis-inducing drugs [16,23]. The super-repressor form of IκBα, as well as a dominant negative form of IKK, also blocked Ras-mediated transformation of cells [24,25] and expression of the IκB super-repressor inhibited anchorage independent growth and metastatic spread of human lung cancer cell lines in a tumor xenograft model [26]. Furthermore, Akt can activate the transcriptional potential of the p65/RELA subunit of NF-κB [27,28], providing a potential link between Akt kinase activity and NF-κB activation. Western blot analysis has demonstrated over expression of the p50 subunit of NF-κB in lung cancer [29], but localization of NF-κB family members has not been described in lung tumors. Nevertheless, abundant evidence links NF-κB transcriptional activation with lung tumorigenesis.

Several NF-κB-regulated genes that function in control of apoptosis have been described including cIAP-1, cIAP-2, A1/Bfl1, Traf1, Traf2 and Bcl-X_L _[30-33]. cIAP-1 and -2 are members of the baculoviral IAP repeat-containing (BIRC) gene family. cIAP-2/BIRC3 is expressed in lung adenocarcinoma cell lines [34] and can be induced by TNF-α [35]. Elevated expression of cIAP-2/BIRC3 has been reported in human NSCLC [36-38] and elevated expression of the related proteins XIAP/BIRC4 and survivin/BIRC5 are also seen in NSCLC [36,39], implicating the BIRC family of proteins as important mediators of lung tumorigenesis.

While the expression of pro-survival genes has been examined in some detail in lung tumors, relatively few studies have examined the expression of these proteins in pre-neoplastic lesions. To determine the presence and abundance of pro-survival proteins in developing lung neoplasia, we examined human bronchial biopsies of various grades (normal, hyperplasia, mild, moderate and severe dysplasia, carcinoma in situ and carcinoma) for the presence of phospho-Akt, p65/RELA and cIAP-2/BIRC3 and determined their localization within cells. The results obtained provide new insight into the distribution of pro-survival genes in human lung neoplasia and pre-neoplasia. This information may be useful in designing studies to test the importance of these pathways experimentally and ultimately may lead to improved diagnostic or therapeutic strategies for human lung cancer.

## Methods

### Description of samples

Bronchial biopsies were obtained during autofluorescence [40] bronchoscopic examination, formalin fixed and embedded in paraffin. Five-micron sections were stained with H&E, examined and scored for pathology grade by an experienced lung pathologist (JCL) using the WHO classification [41]. A separate group of surgically resected NSCLC samples (adenocarcinoma and squamous cell carcinoma) was obtained from Surgical Pathology at the University of Cincinnati. All human samples were obtained under approved IRB protocols at the respective institutions. Table 1 describes the general properties of the biopsies stained including age and gender. Age and gender distribution among the groups was not statistically significant (one way ANOVA, p = 0.558 for age; Kruskal-Wallis one way ANOVA on ranks, p = 0.158 for gender). Of the 22 carcinomas where staging data was available, 36% were stage IA, 23% stage IB, 9% stage IIB, 23% stage III or IV and 9% unclassified. Tumor stages were assigned based on pTNM classifications according to UICC guidelines [42].

**Table 1:** 

**Pathology Grade**	**No. patients**	**No. biopsies**	**Median age**	**Gender (% female)**
Normal	10	12	62.7	70
Hyperplasia	11	11	64.3	45
Mild/Moderate Dysplasia	17	23	64.2	53
Severe Dysplasia/CIS	7	9	67.9	29
Carcinoma	22	24	60.5	77

### Antibodies and Immunohistochemistry

Rabbit polyclonal antibodies for phosphorylated (Ser473, catalog # 9277) and total Akt (catalog # 9272) were purchased from Cell Signaling Technologies (Beverley, MA) and were both used at 1:100 dilution. The p65/RELA antibody (catalog # ab7970) was purchased from Abcam America (Cambridge, MA) and used at a 1:4000 dilution. The cIAP-2/BIRC3 antibody (catalog # AF817) was from R&D Systems (Minneapolis, MN) and used at a 1:500 dilution. Biotinylated goat anti-rabbit secondary antibody and normal goat serum was from Vector Laboratories (Burlingame, CA).

Paraffin embedded sections were deparaffinized through xylene and a graded series of ethanols. Endogenous peroxidase activity was quenched by incubation in 2% hydrogen peroxide in methanol for 15 minutes then cleared in PBS for 5 minutes. High temperature antigen unmasking in citrate buffer was used for all antibodies and carried out as described previously [43]. Non-specific binding was blocked by incubation with 5% normal goat serum in PBS + 0.2% Triton X-100 (blocking serum) for 2 hours at room temperature. Slides were then incubated overnight at 4°C with primary antibody at the appropriate dilution in blocking serum. The next day slides were washed 5× for 5 minutes each in PBS + 0.2% Triton X-100 before addition of secondary antibody. Secondary antibody was added at a 1:200 dilution in PBS+Triton and incubated for 30 minutes at room temperature with shaking. Slides were then washed 5× for 5 minutes each with PBS+Triton. A Vectastain ABC kit (Vector Laboratories) was used to prepare avidin-biotin complexes for detection of secondary antibody. Antigen localization was enhanced with Ni-DAB and Tris-cobalt [44] followed by counterstaining with Nuclear Fast Red.

### Evaluation of immunohistochemical staining and statistical analysis

Biopsies received a numerical score of 0 for negative staining, 1 for predominantly faint staining, 2 for predominantly moderate staining or 3 for predominantly strong staining. For the purpose of this study, the bronchial biopsies were divided into 5 categories for phosphorylated Akt and cIAP-2/BIRC3 analysis: normal, hyperplasia, mild dysplasia or moderate dysplasia, severe dysplasia or carcinoma in situ and carcinoma. For analysis of p65/RELA nuclear translocation, the mild and moderate dysplasia groups were analyzed separately as there was a large difference in the percentage of nuclear staining between these two groups. No significant difference in staining intensity between mild and moderate dysplasia was seen for phosphorylated Akt or cIAP-2/BIRC3. Statistical analysis was performed using SigmaStat software (SyStat Software, Inc.). Analysis of variance for each stain was calculated using a Kruskal-Wallis chi-squared test. Pairwise comparisons were calculated using a non-parametric Mann-Whitney rank-sum test. A p-value < 0.05 was considered significant.

## Results

### Immunohistochemistry for Akt protein kinase

**Figure 1:**
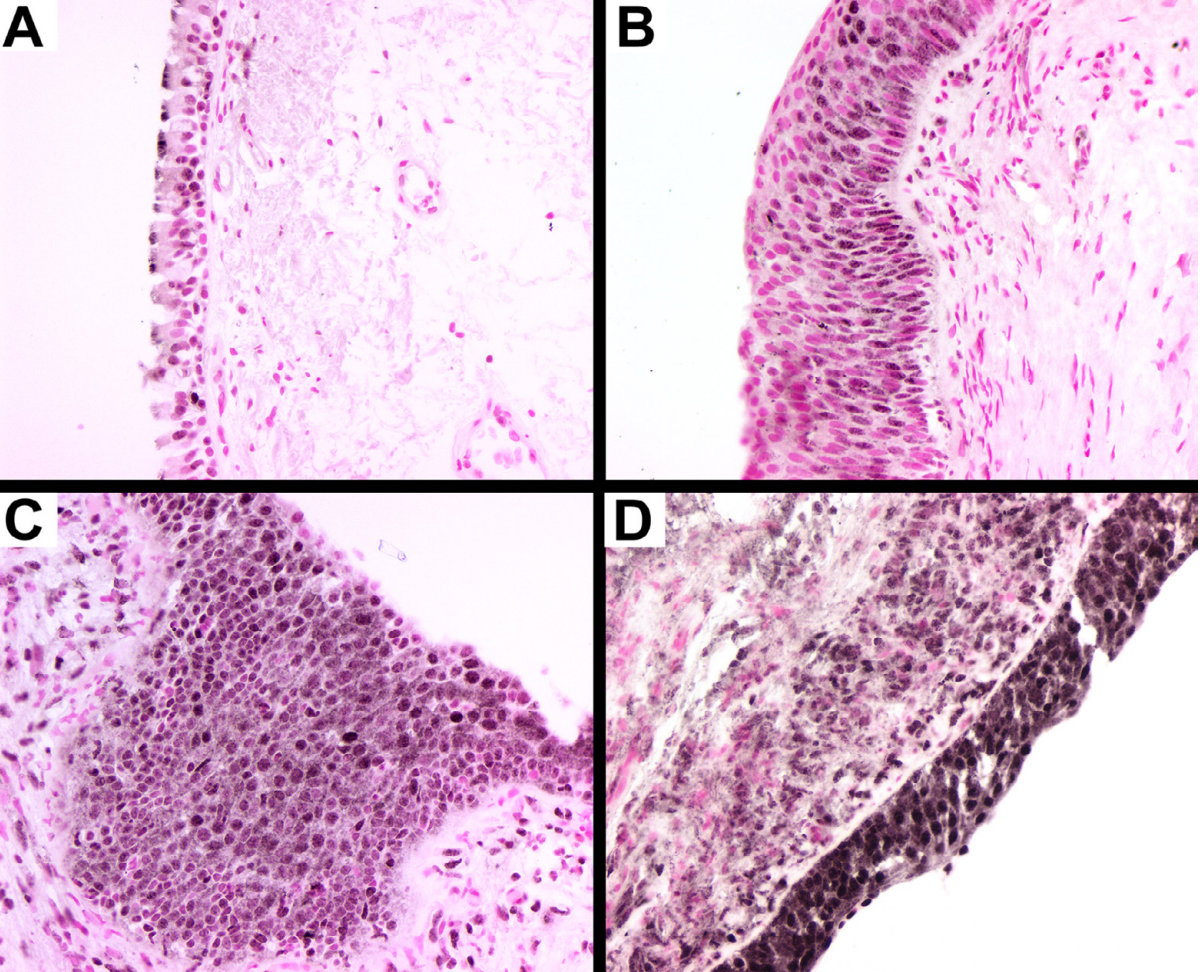
**Immunohistochemical localization of phospho-Akt in human bronchial biopsies**. Sections from human bronchial biopsies were incubated with antibodies specific for the phosphorylated form (ser473) of Akt, color developed with nickel-DAB (black) and counterstained with nuclear fast red. Representative stains of normal (A), mild dysplasia (B), moderate dysplasia (C) and severe dysplasia (D).

**Figure 2:**
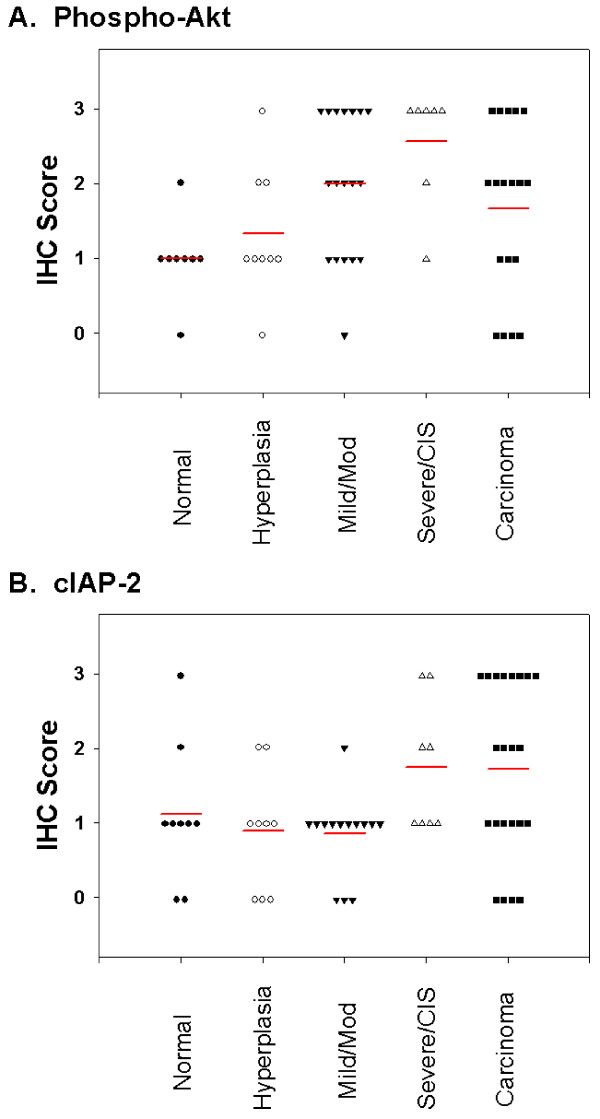
**Frequency plots of predominant IHC score for phospho-Akt and cIAP-2/BIRC3 in human pre-neoplastic bronchial biopsies**. Pre-neoplastic biopsies were scored based on the predominant staining intensity observed using a scale of 0 for negative, 1 for faint, 2 for moderate and 3 for strong. Each symbol represents a separate biopsy and the horizontal line represents the mean score for each category: normal, hyperplasia, mild or moderate dysplasia, severe dysplasia or carcinoma in situ and carcinoma. (A), Scores for predominant phospho-Akt staining intensity. Mean scores were: normal, 1.0; hyperplasia, 1.3; mild or moderate dysplasia, 2.0; severe dysplasia or carcinoma in situ, 2.6; carcinoma, 1.6. (B), Scores for predominant cIAP-2/BIRC3 staining intensity. Mean scores were: normal, 1.1; hyperplasia, 0.89; mild or moderate dysplasia, 0.86; severe dysplasia or carcinoma in situ, 1.8; carcinoma, 1.8.

**Figure 3:**
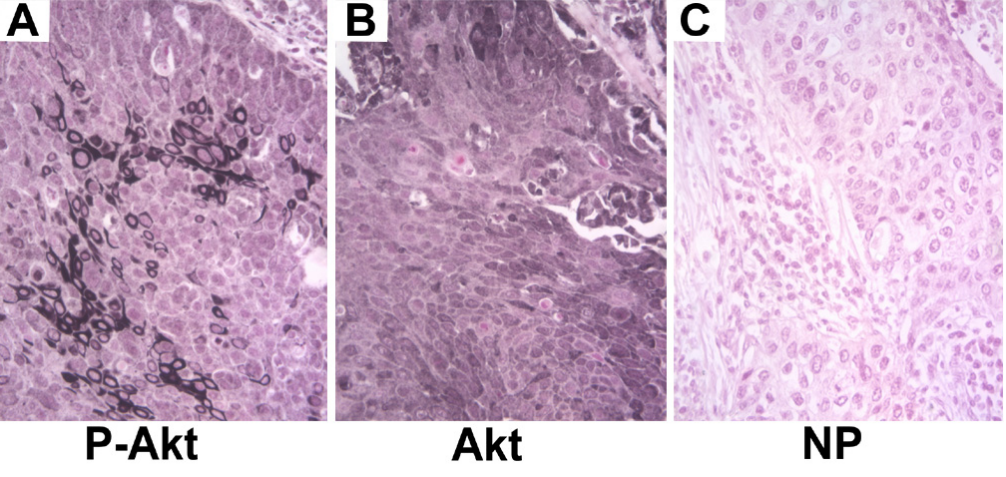
**Immunohistochemical localization of phospho-Akt and total Akt in human squamous cell carcinoma**. Serial section from human squamous cell carcinoma was stained with antibodies specific for phospho-Akt (ser473) (A), or an antibody that recognizes both phosphorylated and non-phosphorylated forms of Akt (B). Localization of phospho-Akt to the plasma membrane of a subset of tumor cells is apparent. Incubation without primary antibody (C) was used as a negative control.

The serine-threonine kinase Akt is a central regulator of cell survival and apoptosis that is itself activated by phosphorylation. Antibodies that specifically recognize the phosphorylated form (Ser 473) were used to identify the phosphorylated, and thus activated, form of Akt. Intensity of staining was scored on a scale of 0 for negative, 1 for predominantly faint staining, 2 for predominantly moderate staining and 3 for predominantly strong staining. In normal bronchial epithelium, phospho-Akt staining typically consisted of faint cytoplasmic stain with occasional cells staining with a more intense nuclear pattern (Figure 1A). In some biopsies, there was a concentration of staining at the apical surface of the epithelium. The underlying interstitial cells were predominantly negative. With increasing pathological grade, there was an overall increase in the intensity of phospho-Akt staining (Figure 1) and an increase in the IHC score (Figure 2A). As pathology grade increased to mild, moderate and severe dysplasia, there were also an increased number of cells with nuclear staining for phospho-Akt (Figure 1B,1C and 1D) in addition to an increase in cytoplasmic staining intensity (analysis of variance analysis using the Kruskal-Wallis Chi-square test, p = 0.018). In addition, pairwise comparisons using the non-parametric Mann-Whitney rank-sum test indicated that staining intensity of the mild/moderate dysplasia and severe dysplasia/CIS groups were significantly increased compared to normal (p = 0.02 and 0.004, respectively). Similar to what has been previously reported [12], invasive carcinoma had a lesser degree of staining than advanced pre-neoplastic lesions (Figure 2A). In squamous cell carcinomas, several tumors had intense plasma membrane associated staining for phospho-Akt (Figure 3A), while an antibody that recognized both phosphorylated and unphosphorylated Akt showed equal staining throughout the cytoplasm and nucleus (Figure 3B).

### Immunohistochemistry for NF-κB subunit p65/relA

**Figure 4:**
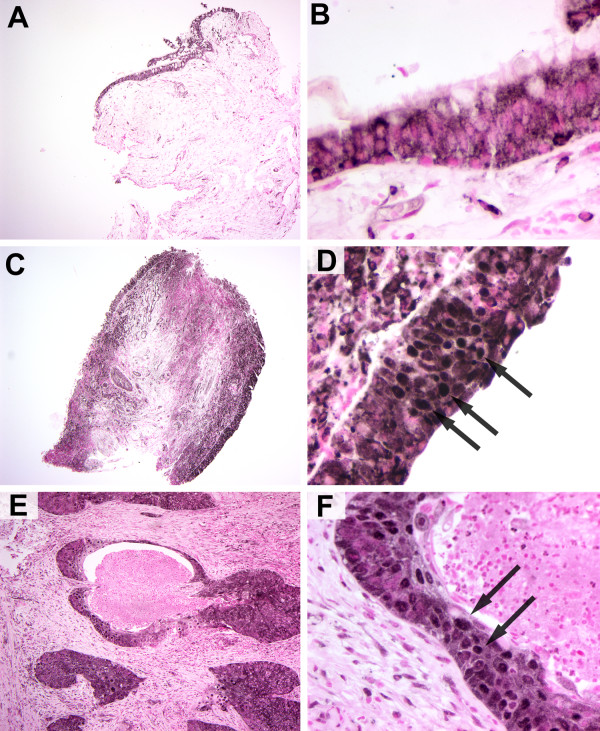
**Nuclear translocation of p65/RELA in progressing lung neoplasia**. Photomicrographs of normal bronchial epithelium (A, B), moderate dysplasia (C, D), or squamous cell carcinoma (E, F) at low (A, C and E) or high (B, D and F) magnification. Staining is diffusely cytoplasmic in normal bronchial epithelium. Nuclear translocation of p65/RELA, rarely detected in normal bronchial epithelium, is present in an increased percentage of moderate to severe dysplasias and squamous cell carcinoma (arrows, D and F).

The transcription factor NF-κB is activated following Akt phosphorylation via several mechanisms and is in itself known to play a role in cell survival and protection from apoptosis. We thus characterized the immunohistochemical staining pattern of the NF-κB subunit p65/RELA in pre-neoplastic bronchial biopsies. NF-κB subunits are normally held in the cytoplasm, thus preventing them from activating transcription, and are translocated to the nucleus following various activation signals such as oxidative stress or growth factor/cytokine stimulation. We therefore determined the staining pattern of p65/RELA in the cytoplasmic or nuclear compartment of airway epithelial cells. Faint to moderate cytoplasmic staining for p65 was present in the cytoplasm of epithelial cells in normal biopsies with little staining in the underlying stroma (Figure 4A and 4B). However, with the exception of one isolated cluster of cells in a single biopsy, p65/RELA staining was not present in the nucleus. With increasing pathology score, nuclear p65/RELA staining in the bronchial epithelium was seen in an increasing percentage of biopsies (Table 2). There was also a trend towards increased intensity of cytoplasmic staining with increasing pathology grade (Figure 4). Analysis of variance gave a significant p-value of 0.010 for differences in percent nuclear localization of p65 among the groups. Pairwise comparisons indicated that moderate dysplasia and severe dysplasia/CIS were significantly different from normal (p = 0.022 and 0.048, respectively). In normal bronchial epithelium, staining of the submucosa was restricted to a few isolated cells, however, many biopsies with moderate to severe dysplasia showed intense staining in submucosal cells in addition to intense epithelial staining (Figure 4C and 4D). In NSCLC, nuclear localization of p65/RELA was seen more frequently in squamous cell carcinomas (Figure 4E and 4F) then adenocarcinomas. In all cases, nuclear p65/RELA staining was focal within a biopsy.

**Table 2:** Percent of biopsies with nuclear p65/RELA staining.

Pathology Grade	**% nuclear p65/RELA stain**
Normal	11 (n = 9)
Hyperplasia	20 (n = 10)
Mild Dysplasia	10 (n = 10)
Moderate Dysplasia	73 (n = 11)
Severe dysplasia/CIS	71 (n = 7)
Carcinoma	41 (n = 17)

### Immunohistochemistry for cIAP-2

**Figure 5:**
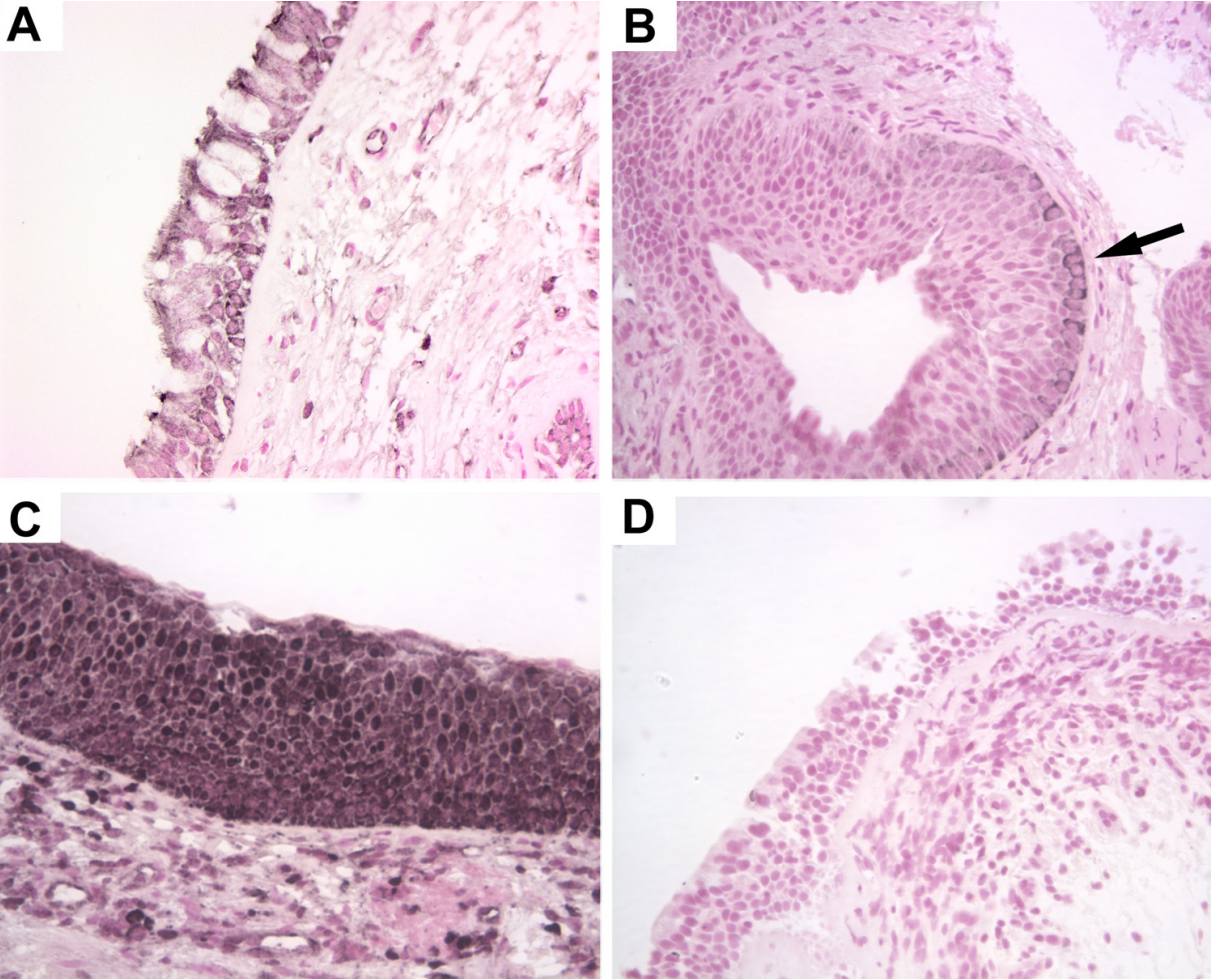
**Immunohistochemical localization of cIAP-2/BIRC3**. Photomicrographs of normal bronchial epithelium (A), mild dysplasia (B), severe dysplasia (C) and a negative control processed without primary antibody (D). In normal and mild dysplasia, perinuclear staining was observed, predominantly in cells adjacent to the basement membrane (for example, arrow in panel B). Increased staining intensity and increased frequency of nuclear staining was observed in severe dysplasias (panel C).

The inhibitor of apoptosis protein (IAP) family function to block apoptosis by caspase-dependent and caspase-independent means [45]. Several members of this family, including cIAP-2, are known transcriptional targets of NF-κB [31]. In normal bronchial epithelium, cIAP-2 staining was typically present in a faint cytoplasmic pattern (Figure 5A). In some biopsies, a perinuclear pattern was observed in epithelial basal cells (Figure 5A and 5B) and there were scattered cells with positive nuclear stain. The average intensity and localization of staining did not differ significantly from normal in hyperplasias or in mild to moderate dysplasias (Figure 2B). Staining intensity was increased in the severe dysplasia/CIS category and in carcinomas (Figure 5C). Strong perinuclear and nuclear staining was common and more widespread in the higher pathology grades compared to normal. Staining of cells in the submucosa was also seen more frequently in the higher pathology grades (severe dysplasia/CIS and carcinoma) compared to normal (Figure 5C). Analysis of variance on cIAP-2 staining intensity indicated a significant difference among the groups (p = 0.048).

### Correlation between cIAP-2 and phospho-Akt staining intensity

We compared staining intensity of phospho-Akt and cIAP-2 in biopsies where both stains were performed on serial sections (n = 53). There was a significant correlation between phospho-Akt and cIAP-2 staining intensities in these biopsies (Spearman correlation coefficient, r = 0.484, p = 0.0002). This correlation was seen at all pathology grades.

**Figure 6:**
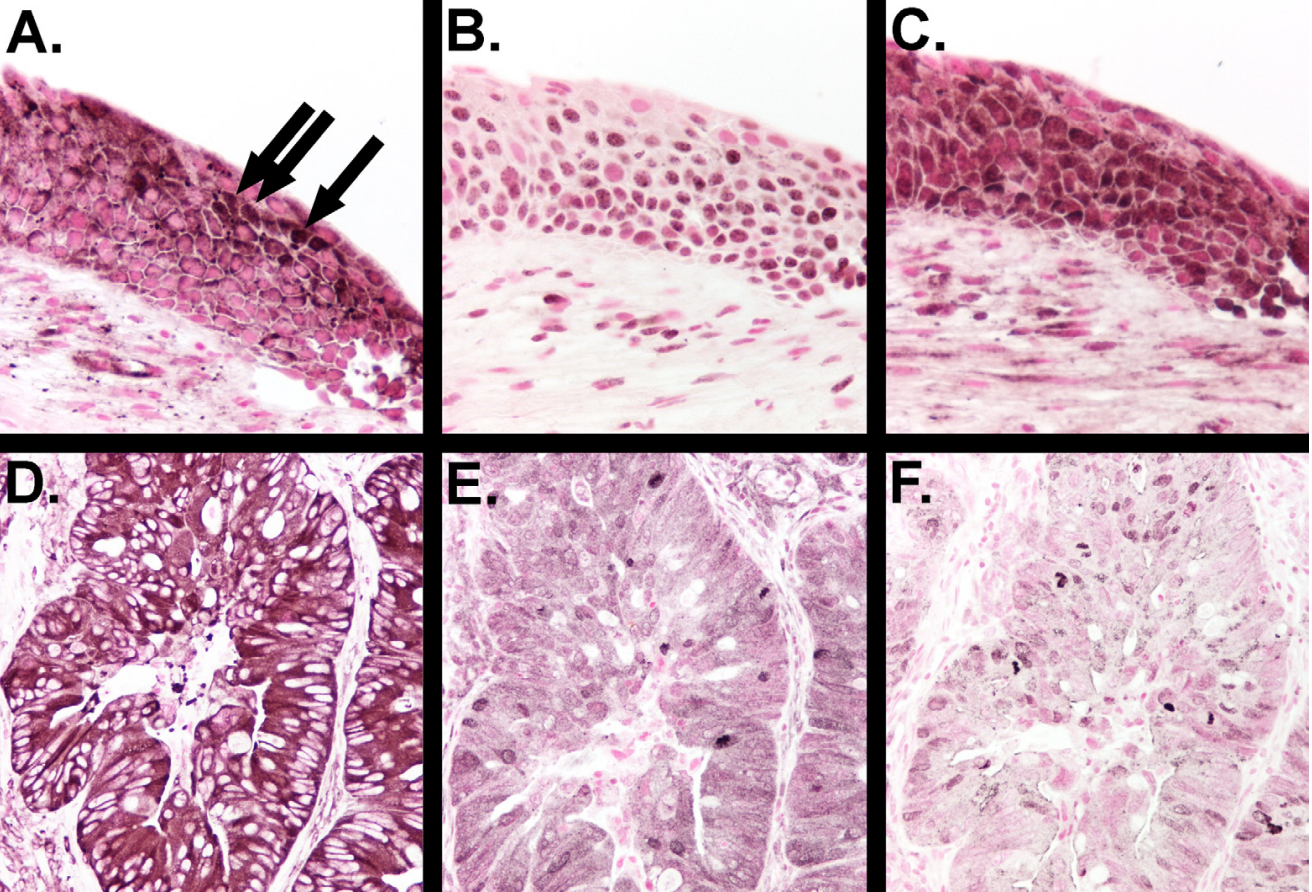
**Staining for p65/RELA, phospho-Akt and cIAP-2 on serial biopsy sections**. Serial sections from a moderate dysplasia (A–C) or adenocarcinoma (D–F) underwent immunohistochemical staining with antibodies directed against p65/RELA (A and D), phospho-Akt (B and E) or cIAP-2 (C and F). Cells with nuclear localization of p65/RELA (arrows in A) correlated with regions of increased staining for phospho-Akt and cIAP-2 in this moderate dysplasia but the correlation was not consistently observed. High levels of cytoplasmic p65/RELA staining in the adenocarcinoma corresponded with faint to moderate staining for phospho-Akt and cIAP-2 (D–F). There was a statistically significant correlation between cIAP-2 and phospho-Akt staining intensity at all pathology grades.

The cIAP-2 gene is a transcriptional target for NFκB activation [31]. To examine whether cIAP-2 expression was increased specifically in areas of nuclear p65/RELA staining, we examined serial sections of biopsies stained with both antibodies. AN example of serial sections of a moderate dysplasia (Figure 6A–C) demonstrates nuclear p65/RELA staining accompanied by increased staining intensity of phospho-Akt and cIAP-2. An adenocarcinoma with strong cytoplasmic staining for p65/RELA with moderate staining for phospho-Akt and cIAP-2 is also shown (Figure 6D–F). Co-localization of nuclear p65/RELA staining and increased cIAP-2 staining was not a consistent observation, and elevated cIAP-2 staining was seen in bronchial IEN lesions without detectable nuclear staining for p65/RELA. Additionally, even in biopsies with nuclear localization of p65/RELA, the number of cells with nuclear p65/RELA was fewer than the number of cells with increased cIAP-2 staining intensity. Thus, there was not a clear correlation between nuclear p65/RELA staining and increased staining intensity for cIAP-2 in human bronchial IEN lesions or human NSCLC.

We also examined if there was a correlation between tumor stage and staining intensity or nuclear localization of p65/RELA in carcinomas. No correlation was seen between tumor stage and any of the stains examined.

## Discussion

The pathways utilized by lung tumors to evade apoptosis, allowing the tumors continued survival and growth, are incompletely characterized. Equally important are the pathways that become activated in IEN lesions before they become invasive cancer. In this study, we examined the presence and cellular localization of the phosphorylated form of Akt kinase, the transcription factor p65/RELA and the cellular inhibitor of apoptosis protein cIAP-2/BIRC3 in human bronchial IEN lesions. In normal human bronchial epithelium, staining for phosphorylated Akt was present as a faint cytoplasmic stain. Staining intensity increased with increasing pathology grade and was present in a nuclear, perinuclear or plasma membrane pattern. The intensity of p65/RELA staining also increased with increasing pathology grade. Furthermore, more biopsies had nuclear localization of p65/RELA with advancing pathology grade, indicating nuclear translocation of the protein and the potential for transcriptional activation. The apoptosis inhibitor cIAP-2/BIRC3 also had increased staining intensity with increasing pathology grade. These results indicate that Akt, p65/RELA and cIAP-2/BIRC3, components of an anti-apoptotic pathway, are elevated in pre-neoplastic lung lesions.

The serine/threonine kinase Akt is a central mediator of anti-apoptotic pathways in eukaryotic cells [2]. Activation of this kinase occurs when it is itself activated by PI3K-dependent protein kinase 1 or 2 (PDK1 or 2). Previous studies have noted an increase in the amount of phosphorylated Akt in a number of different human malignancies including cancer of the lung [4,7,12], head and neck [46], prostate [9], and in multiple myeloma [8,47]. In addition, loss of the tumor suppressor phosphatase and tensin homologue (PTEN), a lipid phosphatase that inhibits PI3K activity, is found in a subset of lung tumors [48-50]. Increased staining for phosphorylated Akt was also frequently observed in human bronchial pre-neoplastic lesions [4,7,12]. In bronchial IEN, increased staining for phospho-Akt correlated with increased staining for phospho-FKHR, a transcription factor that directs expression of anti-apoptotic genes in response to Akt signaling [7]. Our present results agree with previous studies demonstrating an increase in phospho-Akt staining in pre-neoplastic human bronchial lesions. We observed increased staining for the phosphorylated form of Akt beginning as early as mild dysplasia lesions in human bronchial biopsies.

The staining for phospho-Akt observed in the biopsies in our study was diverse, consisting of cytoplasmic, nuclear or plasma membrane associated patterns. As PDK must associate with PI3K at the plasma membrane to be activated, localization of Akt has often also been localized to the cytoplasm or plasma membrane [8,9]. However, several targets of Akt are nuclear transcription factors and nuclear localization of Akt has been described in several other human tumor types [7,12,47]. The differences in subcellular localization observed for phospho-Akt in different biopsies may reflect the large number of pathways regulated by this kinase and the different mechanisms by which Akt can mediate its anti-apoptotic effects. The functional significance of the variable localization of phospho-Akt in human lung tumors remains to be determined.

The NF-κB transcription factor family is known to direct both apoptotic and anti-apoptotic signaling in eukaryotic cells [51]. In unstimulated cells, NF-κB transcription complexes are held in the cytoplasm by IκB proteins, thus preventing their function as transcriptional activators in the nucleus. Cytokine or growth factor stimulation initiates a signaling cascade that leads to activation of the IκB kinase (IKK) complex that in turn phosphorylates IκB and targets it for ubiquitination and ultimately degradation by the proteasome. Degradation of IκB allows NF-κB to be translocated to the nucleus where it can activate transcription of target genes. Inhibition of NF-κB blocked Ras-mediated transformation of cell lines [24] indicating NF-κB activity is critical for Ras-mediated transformation. The Akt kinase has been reported to activate NF-κB by phosphorylation of IKK [52], causing degradation of IκB and nuclear translocation of NF-κB complexes, and by directly phosphorylating the p65/RELA subunit of NF-κB and increasing its transcriptional activity [27,28]. It should also be noted that Akt can be activated by NF-κB [53], suggesting the regulatory pathways utilized by these molecules is complex.

While ample evidence has implicated NF-κB activation as playing an important role in cell transformation, particularly in vitro, there is little data on the presence or localization of NF-κB components in human lung tumors. Chemotherapeutic agents induced NF-κB activity in NSCLC cell lines, increasing the cells resistance to these agents [17] suggesting that in addition to a role in tumor cell evasion of apoptosis, NF-κB may render lung tumor cells more resistant to chemotherapeutic agents. The NF-κB subunit p50 was increased in NSCLC as detected by immunoblotting but no information on localization of this protein within tumors was obtained [29]. To our knowledge, no other study has examined the localization of the p65 subunit of NF-κB in lung IEN lesions or NSCLC. We therefore decided to examine human IEN lesions and lung carcinomas for the presence and localization of the p65/RELA subunit of NF-κB.

In our study, nuclear translocation of the p65/RELA subunit of NF-κB was significantly increased in moderate dysplasias compared to lower grade lesions. In the carcinomas, there was a reduction of p65/RELA nuclear positivity similar to the decreased intensity of phospho-Akt staining observed in carcinomas compared to moderate and severe dysplasias. The percentage of cells with positive nuclear staining in carcinomas was also very low compared to moderate and severe dysplasias generally being restricted to isolated foci. In addition to the increased number of biopsies with nuclear staining at higher pathology grades, the overall intensity of cytoplasmic tended to be increased in moderate dysplasias in both the epithelium and stroma compared to normal epithelium (although this difference was not statistically significant in our analysis). In a number of carcinomas, there was intense cytoplasmic staining for p65/RELA with an apparent paucity of staining in the nucleus. While these biopsies were scored negative for nuclear staining of p65/RELA, it was impossible to discount the possibility that nuclear p65/RELA was present in these cells but at levels lower than those observed in the cytoplasm. In the lung adenocarcinoma cell line NCI-H441 we have also observed higher levels of p65 immunoreactivity in the cytoplasm than the nucleus although these cells have very high levels of NF-κB activity as measured by reporter gene assays (JWT and MWA, unpublished data). It should also be noted that activation of NF-κB has been reported in lung cancer cells without an increase in nuclear localization [24,54]. In all lesions with positive nuclear staining, a majority of tumor cells did not contain nuclear p65/RELA staining. The focal nature of nuclear positivity may reflect the induction of this pathway in a subset of cells that has acquired new genetic or epigenetic changes that activate NF-κB and thus may activate downstream anti-apoptotic pathways.

Tumor cells acquire the ability to escape apoptosis that is normally initiated in damaged cells. One mechanism for inhibiting apoptosis is to block the activity of the effector caspases that initiate apoptosis by degrading specific cellular targets [55]. The cellular inhibitor of apoptosis (cIAP) family of proteins, also known as the baculoviral IAP repeat containing (BIRC) family, can inhibit apoptosis by binding effector caspases and blocking their proteolytic activity. Several family members have been described including cIAP-1/BIRC2, cIAP-2/BIRC3, XIAP/BIRC4, survivin/BIRC5 and NAIP/BIRC1, and are thought to be important in tumorigenesis [45]. We chose to examine the levels of cIAP-2/BIRC3 in developing lung neoplasia as it is expressed in lung tissue [38] and is a target for NF-κB transcriptional activation [31].

While increased staining for cIAP-2/BIRC3 in human lung adenocarcinomas has been reported [38], levels of cIAP-2/BIRC3 in IEN lesions in lung epithelium have not been reported. Staining for cIAP-2/BIRC3 was increased in the severe dysplasia/CIS category and in carcinomas compared to less advanced pathology grades. The increase in cIAP-2/BIRC3 staining occurred at a higher pathology grade than the grade at which phospho-Akt staining increased (mild/moderate dysplasias) or p65/RELA nuclear localization was increased (moderate dysplasia). This may indicate that inhibition of apoptosis via this protein is not required until later in lung tumorigenesis. While high levels of cIAP-2/BIRC3 expression correlated with nuclear localization of p65/RELA in some biopsies, this was not a consistent observation. The lack of consistent p65/RELA and cIAP-2/BIRC co-expression, in combination with the observation that the overall increase in cIAP-2/BIRC staining occurred at a later pathological stage than p65/RELA nuclear translocation, may indicate that cIAP-2/BIRC3 is not a direct target of p65/RELA in bronchial pre-neoplasia. The possibility remains that p65/RELA transcriptional activation initiates a program that may indirectly lead to cIAP-2/BIRC3 induction later in the progression of pre-neoplastic bronchial lesions.

While nuclear staining localization of p65/RELA and increased staining for cIAP-2 did not correlate, we observed a positive correlation between staining intensity for cIAP-2 and phospho-Akt. While it is impossible to state whether this indicates that cIAP-2 is regulated by activation of Akt phosphorylation, this is a possibility. It should be noted, however, that staining intensity for phospho-Akt occurred one pathology grade prior to the observed increase in cIAP-2 staining intensity.

## Conclusion

Increased staining for phospho-Akt and cIAP-2/BIRC3 is present in pre-neoplastic human bronchial biopsy lesions compared to normal human bronchial epithelium. Additionally, the percentage of biopsies containing nuclear p65/RELA staining is increased in pre-neoplastic lesions compared to normal bronchial epithelium. The activation of genes involved in protecting cells from apoptosis in pre-neoplastic lesions suggests that the ability of lung epithelial cells to evade apoptosis is acquired prior to the cells becoming fully transformed. As pre-neoplastic lesions have not acquired all the genetic and epigenetic changes present in lung carcinoma, they may be more amenable to treatment with chemopreventive agents. Thus, identification of the pathways activated in human bronchial pre-neoplastic lesions is important in increasing our understanding of how these lesions develop into lung cancer with the eventual goal of targeting these pathways therapeutically.

## Competing interests

The author(s) declare that they have no competing interests.

## Authors' contributions

JWT reviewed histological staining, participated in design of the study and drafted the manuscript. YZ carried out the immunohistochemical analyses. JCL determined pathology grades of bronchial IEN lesions and reviewed histological staining. PWB determined pathology grades of lung carcinomas and helped draft the manuscript. SL participated in the design of the study, obtained IEN lesions and helped draft the manuscript. MWA conceived of the study, participated in study design and helped draft the manuscript. All authors read and approved the final manuscript.

## Pre-publication history

The pre-publication history for this paper can be accessed here:


